# GPR55, a G-Protein Coupled Receptor for Lysophosphatidylinositol, Plays a Role in Motor Coordination

**DOI:** 10.1371/journal.pone.0060314

**Published:** 2013-04-02

**Authors:** Chia-Shan Wu, Hongmei Chen, Hao Sun, Jie Zhu, Chris P. Jew, James Wager-Miller, Alex Straiker, Corinne Spencer, Heather Bradshaw, Ken Mackie, Hui-Chen Lu

**Affiliations:** 1 The Cain Foundation Laboratories, Baylor College of Medicine, Houston, Texas, United States of America; 2 Jan and Dan Duncan Neurological Research Institute at Texas Children’s Hospital, Baylor College of Medicine, Houston, Texas, United States of America; 3 Department of Pediatrics, Baylor College of Medicine, Houston, Texas, United States of America; 4 Program in Developmental Biology, Baylor College of Medicine, Houston, Texas, United States of America; 5 Department of Neuroscience, Baylor College of Medicine, Houston, Texas, United States of America; 6 Department of Genetics, Baylor College of Medicine, Houston, Texas, United States of America; 7 Department of Psychological and Brain Sciences, Indiana University, Bloomington, Indiana, United States of America; Louisiana State University Health Sciences Center, United States of America

## Abstract

The G-protein coupled receptor 55 (GPR55) is activated by lysophosphatidylinositols and some cannabinoids. Recent studies found prominent roles for GPR55 in neuropathic/inflammatory pain, cancer and bone physiology. However, little is known about the role of GPR55 in CNS development and function. To address this question, we performed a detailed characterization of GPR55 knockout mice using molecular, anatomical, electrophysiological, and behavioral assays. Quantitative PCR studies found that GPR55 mRNA was expressed (in order of decreasing abundance) in the striatum, hippocampus, forebrain, cortex, and cerebellum. GPR55 deficiency did not affect the concentrations of endocannabinoids and related lipids or mRNA levels for several components of the endocannabinoid system in the hippocampus. Normal synaptic transmission and short-term as well as long-term synaptic plasticity were found in GPR55 knockout CA1 pyramidal neurons. Deleting GPR55 function did not affect behavioral assays assessing muscle strength, gross motor skills, sensory-motor integration, motor learning, anxiety or depressive behaviors. In addition, GPR55 null mutant mice exhibited normal contextual and auditory-cue conditioned fear learning and memory in a Pavlovian conditioned fear test. In contrast, when presented with tasks requiring more challenging motor responses, GPR55 knockout mice showed impaired movement coordination. Taken together, these results suggest that GPR55 plays a role in motor coordination, but does not strongly regulate CNS development, gross motor movement or several types of learned behavior.

## Introduction

The observation that cannabinoids produce effects independent of CB1 and CB2 cannabinoid receptors motivated the search for additional cannabinoid receptors (reviewed in [1]). The identification of G-protein coupled receptor 55 (GPR55) as a potential cannabinoid receptor generated significant interest [2], [3]. Its potential assignment as a cannabinoid receptor was based on its potent activation by a subset of cannabinoid ligands [1], [4], [5], [6]. Synthetic cannabinoid ligands reported to activate GPR55 include the CB2-preferring aminoalkylindole, JWH015, the CB1 inverse agonists, SR141716A and AM251, and the metabolically stable anandamide analog, methanandamide. Subsequently, lysophosphatidylinositols (LPI) have been identified as endogenous ligands for GPR55 [7]. Thus, GPR55 binds and is activated by an impressive array of structurally diverse ligands. Conversely, all well-characterized and frequently used GPR55 ligands interact with other receptors, channels, and/or signaling molecules at concentrations similar to those that activate or antagonize GPR55 [1], [6]. Further complicating analysis is the considerable variation in experimental results between different laboratories examining GPR55 signaling [8]. GPR55 appears to primarily signal through the G proteins, Gq and Gα_12/13_ [5], [9]. Downstream signaling pathways of GPR55 include: release of calcium from intracellular stores, stimulation of ERK1/2 phosphorylation, activation of Rho kinase, the small G proteins: RhoA, cdc42, and rac1, and stimulation of several transcriptional networks, including those mediated by NFAT, NFκB, and CREB [5], [9], [10].

The absence of potent, rigorously validated GPR55-specific agonists and antagonists makes it very challenging to use a pharmacological approach to determine the endogenous function(s) of GPR55. An alternative is to examine mice lacking GPR55. Studies with GPR55 loss-of-function mutant mice or cultured cells suggested a role for GPR55 in pain [11], bone physiology [12], cancer [13], as well as glucose homeostasis [14]. In a neuropathic pain model, GPR55 null mice failed to develop inflammatory mechanical hyperalgesia in contrast to their wild type littermate controls [11]. GPR55 has also been shown to suppress osteoclast formation, while stimulating osteoclast function through Rho and ERK signaling [12]. In addition, the volume and thickness of long bones were significantly increased in GPR55 null mice. GPR55 activation also enhanced cancer cell mitogenic activity and metastases [15]. Clearly, GPR55 is an exciting, novel, and underexplored receptor around which it may be possible to develop therapies for neuropathic pain [16], osteoporosis [17], [18], cancer [13], [19], [20], and obesity [21].

To reveal potential physiological roles of GPR55 in CNS function, we first examined the abundance of GPR55 in specific brain regions and then examined anatomical structures from GPR55 knockout brains. Next, a panel of electrophysiological and behavioral assays was employed to examine synaptic function and plasticity, as well as behaviors using GPR55 null mutant mice and their littermates. These studies suggest that GPR55 plays a role in regulating motor coordination and pain sensations.

## Materials and Methods

### Animals

GPR55 knockout (KO) mice were acquired from TIGM (Texas A&M Institute for Genomic Medicine) [22]. Mouse colonies were maintained in a pathogen-free environment with a 14-10 h light:dark cycle (lights on at 6 AM) with access to food and water *ad libitum*. The animals used for testing were backcrossed onto the C57BL/6 strain for 8 generations. For all experiments, heterozygous females and heterozygous males were mated to generate GPR55 KO and wild-type (WT) littermate controls. All genotypes were generated in numbers close to the expected Mendelian ratios. After weaning, two to four mice were housed together. Animal procedures have been approved by the Institutional Animal Care and Use Committee of Baylor College of Medicine (protocol # AN-3327) and Indiana University (protocol # 10-004) and were conducted in compliance with the U.S. Department of Health and Human Services (assurance #: 3823-01 for BCM and 4094-01 for IU).

### Genotyping

Animals were genotyped as previously described [22]. Briefly, a mixture of two pairs of primers was used. For detection of the transgene, the following pair 5′ – GCA GCG CAT CGC CTT CTA TC –3′ and 5′ – TCA AGC TAC GTT TTG GGT T –3′ (expected PCR product size is 301 bp) were used. For detection of WT allele, the following pair 5′ – GCC ATC CAG TAC CCG ATC C –3′ and 5′ – GTC CAA GAT AAA GCG GTT CC –3′ (expected PCR product size is 441 bp) were used. PCR cycle conditions were: 5 min at 95°C, 36 cycles of three steps (50 sec at 94°C, 40 sec at 55°C, and 40 sec at 72°C), then 5 min at 72°C using standard PCR reagents.

### Tissue Processing

Mice used for quantitative real-time RT-PCR (qPCR) were sacrificed at five months of age (WT, n = 4; GPR55 KO, n = 6). Anesthetized mice were decapitated, tissue harvested and immediately placed into RNAlater (Ambion, Austin, TX) and stored as per manufacturer’s instructions. Prefrontal cortex was taken to be the anterior 3 mm of cortex, and “cortex,” refers to the remaining cortex, minus the hippocampus. Mice at 2 months of age were processed for histology and immunohistochemistry. Mice were deeply anesthetized with an intraperitoneal injection (3 ml/kg) of a rodent anesthetic cocktail containing ketamine 37.6 mg/ml, xylazine 1.92 mg/ml and acepromazine 0.38 mg/ml. Following establishment of anesthesia, mice were transcardially perfused with PBS, pH 7.4, followed by 4% PFA in PBS, pH 7.4. The brains were then post-fixed with the same fixative overnight at 4°C, washed with PBS and stored in 0.1% sodium azide/PBS at 4°C until processing.

### Quantitative Polymerase Chain Reaction Analysis

Primers for selected components of the endocannabinoid system were designed using Primer-Blast (http://www.ncbi.nlm.nih.gov/tools/primer-blast) and the corresponding mouse gene. The following mixtures of two primers was used for detection of cDNAs: for ABHD6, 5′–CCT TGA TCC CAT CCA CCC CGG A–3′ and 5′–CCC GGA CAC ATC AAG CAC CTG G–3′; for DGLα, 5′– CAC AGA GGC ACC TGG TTG GGC–3′ and 5′– TCC GCC ATT TGG GCT TGG TGC–3′; for DGLβ, 5′–GGC GAC TGT TGC AGA GCC AGA G–3′ and 5′–GCA TGA TGG CTA ACA GGG CCG C–3′; for FAAH, 5′–GCC TCT GTT TCC TCG GCT GGC–3′ and 5′–ACC CCC GCA GGG AAG TCC AG–3′; for MGL, 5′–TCT TCC TCC TGG GCC ACT–3′ and 5′–AAA GTA GGT TGG CCT CTC TGC–3′.

Tissues stored in RNAlater were dried and RNA was extracted using Tri reagent (Ambion, Austin, TX) and genomic DNA was removed with DNase (NEB, Bethesda, MD) following the manufacturer’s instructions. RT-PCR was performed using a one-step, Sybr Green amplification process (PwrSybr, Applied Biosystems, Carlsbad, CA).

Quantitative PCR was performed using a Stratagene Mx3000P thermocycler. Primers for glutaraldehyde-3-phosphate dehydrogenase (GADPH) or β-actin were used as internal controls for each brain region with the threshold cycle set within the linear range (10 fold above baseline). Once the standard critical threshold (Ct) was set, the relative expression levels for genes for each brain region were determined. Data analysis and statistics were performed using Excel (Microsoft Corp., Redmond, WA) and Prism (GraphPad Software Inc., San Diego, CA) software.

### Histology and Immunohistochemistry

Brains were serially sectioned in the coronal or sagittal plane into 100 µm thick sections using a Leica VT-1000 vibrating microtome (Leica Microsystems, Bannockburn, IL). One set of sections was mounted on slides, air-dried overnight, and subjected to Nissl staining. For immunohistochemical staining, free-floating sections were washed with PBS/0.01% Triton X-100 (PBST) and permeabilized with 0.2% Triton X-100 in PBS at room temperature for 20 minutes. Free-floating sections were then incubated with 30% methanol and 3% H_2_O_2_ in PBST, washed extensively with PBST and blocked for one hour with 3% normal goat serum in PBST at room temperature. Sections were then incubated with primary antibodies diluted in PBST/1% normal goat serum/2% BSA at 4°C overnight. The next day, sections were washed with PBST, and incubated with biotin-conjugated goat anti-rabbit antibodies (1∶500, Jackson ImmunoResearch, West Grove, PA) in PBST at room temperature for two hours. Following this incubation, sections were washed with PBST three times for 10 minutes each, then incubated with avidin/biotin complex (Vectastain ABC kit, Vector Labs, Burlingame, CA) for two hours at room temperature. Sections were then washed, and immuno-signals detected with a DAB kit (Vector Labs). Color development was observed under a dissecting microscope. Sections were washed with PBS, mounted, and air-dried overnight before counterstaining with Nissl. Immunofluorescence staining was similar to the procedure described above, with the following modifications: the quenching step with hydrogen peroxide was omitted, and Alexa 488-conjugated goat anti-rabbit antibodies and Cy3-conjugated goat anti-mouse antibodies (all at 1∶500, Invitrogen) were used. Sections were counterstained with DAPI and mounted with Vectashield (Vector Labs). The following primary antibodies were used: rabbit anti-tyrosine hydroxylase (1∶2000, Millipore, Billerica, MA), rabbit anti-vesicular glutamate transporter 2 (VGluT2, 1∶1000, Synaptic Systems) and mouse anti-NeuN (1∶1000, Millipore). Bright field images were taken with an Olympus BX51 upright microscope under consistent light conditions using an Olympus DP70 CCD camera with Olympus DPC controller software with 2x/0.08 Plan Apo and 4x/0.16 U Plan Apo objectives (magnification/numerical aperture). Fluorescent images were taken with a Zeiss AxioImager M1 system with 5x/0.16 Zeiss objectives, using AxioVision software. All images were processed in Adobe Photoshop CS2 for brightness/contrast, orientations, and background corrections to better illustrate the staining patterns.

### Endocannabinoid Quantification

Anadamide-d_4_ was purchased from Tocris Bioscience (St. Louis, MO). N-arachidonoyl ethanolamide (Anandamide; AEA), 2-arachidonoyl glycerol (2-AG), 2-linoleoyl glycerol (2-LG), N-oleoyl ethanolamide (OEA), N-palmitoyl ethanolamide (PEA), N-linoleoyl ethanolamide (LEA), N-arachidonoyl glycine (NAGly), N-palmitoyl glycine (PalGly), N-arachidonoyl serine (AraSer), N-oleoyl serine (OlSer), Prostaglandin E2 (PGE2), and Prostaglandin F2α (PGF2α) were purchased from Cayman Chemical (Ann Arbor, MI). HPLC-grade water and methanol were purchased from VWR International (Plainview, NY). HPLC-grade acetic acid and ammonium acetate were purchased from Sigma-Aldrich (St. Louis, MO).

Lipid extracts of tissues were performed as previously described [23]. Samples were separated using a C18 Zorbax reversed-phase analytical column. Gradient elution (200 µL/min) was driven using two Shimadzu 10AdVP pumps (Columbia, MD). Eluted samples were analyzed by electrospray ionization using an Applied Biosystems/MDS Sciex (Foster City, CA) API3000 triple quadrupole mass spectrometer. A multiple reaction monitoring (MRM) setting on the LC/MS/MS was then used to analyze levels of each compound present in the sample injection. Synthetic standards were used to generate optimized MRM methods and standard curves for analysis.

### Hippocampal Cell Culture and Electrophysiology

Hippocampal neurons isolated from the CA1–CA3 region of WT or GPR55 knockout P1–P2 mouse pups were cultured for 8–10 days on microislands and recordings performed as previously described [24]. In these neurons increasing the duration of depolarization stimulus results in progressively stronger inhibition of excitatory postsynaptic currents (EPSCs) via a CB_1_ cannabinoid receptor-sensitive depolarization-induced suppression of excitation (DSE) [24]. We have found it is convenient to use depolarization duration (in seconds) as a “dose”, plotted on a log scale to obtain a log “stimulus duration-response curve” with properties similar to a classical dose response curve. Taking the largest maximal slope of the curve in combination with observed baseline and maximal responses, allows us to derive an ‘effective dose’ (ED-50). This ED-50 reflects the duration of depolarization required to induce a response halfway between the baseline and the maximum response. Relative EPSC charge data are presented as proportions (relative to baseline). Non-linear regression was used to fit the concentration response curves. Treatment effects were evaluated by examining for overlap of 95% confidence intervals (95% CI).

### Hippocampal Slice Electrophysiology

All data acquisition and analysis was carried out blinded to genotype. For field potential recordings, 3 month-old mice were anesthetized with isoflurane and their brains were removed and immersed immediately in ice-cold cutting solution (in mM): 220 sucrose, 2.5 KCl, 0.5 CaCl_2_, 7 MgCl_2_, 1.25 NaH_2_PO4, 25 NaHCO_3_, 7 D-glucose saturated with 95% O_2_ and 5% CO_2_. 400 µm thick transverse hippocampal slices were prepared with a vibrating microtome Series 1000 (Vibratome Company, St. Louis, MO) and recovered at 31±0.5°C for an hour in an interface chamber. Field excitatory postsynaptic potential (fEPSP) were recorded at 31±0.5°C in an interface chamber perfused at 1 ml/min with artificial cerebrospinal fluid (ACSF) containing (in mM): 125 NaCl, 2.5 KCl, 2 CaCl_2_, 1 MgCl_2_, 1.25 NaH_2_PO_4_, 25 NaHCO_3_, and 14.83 D-glucose. Extracellular stimuli were administered along the Schaffer collaterals using Formvar-insulated, bipolar nichrome electrodes controlled by a stimulus isolator (A-M Systems, Carlsborg, WA). An ACSF-filled glass-recording electrode was placed in *stratum radiatum* to record the field potential changes. Electrophysiological traces were amplified with AC-coupled amplifier (model 1800; A-M Systems), digitized using a Digidata 1320A (Molecular Devices, Union City, CA), and acquired with pClamp 10 software (Molecular Devices).

To assess baseline synaptic transmission, input-output relationships were examined by measuring the rising slope of the fEPSP evoked by 100 µs pulses over various stimulus intensities (1 V to 10 V). The stimulation intensity that evoked a fEPSP whose slope was 30–40% of the maximum fEPSP slope, determined by the input-output recording experiment, was used for the following recording paradigms. A paired-pulse recording paradigm was conducted with paired stimuli separated with inter-stimulus intervals of 10, 20, 50, 100, or 200 ms. Stimulus pairs were delivered at 0.05 Hz and five trials were averaged at each inter-stimulus interval given in a random order. The paired-pulse ratio was calculated by dividing the slope of the second fEPSP by the slope of the first fEPSP. Long-term potentiation (LTP) was induced by either a theta burst stimulation (TBS) consisting of 10 trains of five stimuli at 100 Hz, repeated 5 times with an inter-train interval of 5 sec, or a high-frequency stimulation (HFS) protocol, which consisted of 2 trains of 100 pulses at 100 Hz that were 20 sec apart [25]. To monitor LTP development, the fEPSPs were recorded every 20 seconds for 20 minutes before and 60 minutes after induction. The magnitude of potentiation was determined by measuring the changes in fEPSP slope. All data are shown as mean ± SEM. Input-output and paired-pulse ratio data were analyzed as two-way ANOVA with repeated measures for each parameter. TBS- and HFS-LTP at 16–20 min time bin were analyzed by Student’s t-test.

For whole-cell patch-clamp recordings, acute hippocampal slices were prepared from 6–7 week old male mice. Brains were removed and immersed immediately in oxygenated ice-cold cutting solution (in mM): 110 choline-chloride, 25 NaHCO_3_, 25 D-glucose, 11.6 sodium ascorbate, 7 MgSO_4_, 3.1 sodium pyruvate, 2.5 KCl, 1.25 NaH_2_PO_4_, and 0.5 CaCl_2_. Brains were sectioned in a coronal plane at 350 µm thicknesses using a Leica VT-1000 vibrating microtome. During recording, slices were maintained in recording chamber with 32–33°C oxygenated ACSF perfused at 2 ml/min. Voltage-clamped spontaneous EPSCs (sEPSCs) were recorded from CA1 pyramidal neurons using a Multiclamp 700B amplifier (Molecular Devices) at 10 kHz sampling rate with 4 kHz Bessel filter using pClamp 10. High-seal (GΩ) and low-series (<30 MΩ) resistances were monitored throughout the experiment to ensure high-quality recordings.

Spontaneous EPSCs were recorded at −60 mV. Recording pipettes (4–7 MΩ tip resistance) made from borosilicate glass were filled with intracellular solution (containing the following in mM: 117.5 potassium gluconate, 17.5 KCl, 8 NaCl, 10 HEPES, 0.2 EGTA, 4 Mg-ATP, 0.3 GTP, and 7 phosphocreatine, pH 7.2, 290–300 mOsm). Five-minute recording traces were analyzed for their frequency and amplitude distributions using MiniAnalysis (Synaptosoft, Decatur, GA). The root mean square (RMS) of the background noise was computed for each set of data. The detection threshold for an event was set to three times the RMS value.

Current-clamped whole-cell recordings were also conducted to examine excitability and membrane properties. A series of current injection steps (range: −100 pA to +400 pA with 50 pA per step) were given to evoke action potentials (APs). Current pulse durations were 600 ms for AP train recording. All summary data are presented as means ± SEM. Student’s t-test was used to determine statistical significance. Prism 3.0 (Graph-Pad Software) was used for all statistical analyses.

### Behavioral Testing

Male and female mice at 2–5 months of age were subjected to selected tests from the test battery originally described by Crawley and Paylor [26]. Tests were performed in the following order with at least 3 days between tests: elevated plus-maze, open-field, prepulse inhibition (PPI), rotarod, conditioned fear and hotplate. A separate cohort of animals was used in the following assays: footslips, dowel, wire-hang, inverted screen, grip strength, and forced swimming. Prior to any behavioral testing, mice were allowed to acclimatize to the testing room for at least 30 min. Behavioral testing was performed between 10 AM and 4 PM (mid phase of light cycle). Experimenters were blind to genotype.

### Elevated Plus-maze

The elevated plus-maze was made of four perpendicular runways (7×25 cm) elevated 40 cm off the ground. Two arms were enclosed by 15 cm white walls and two arms were open, except for a small 5 mm rim. The test animals were placed in the center of the elevated maze facing one of the two open arms, and left to explore for 10-minutes. The number of entries, distance traveled and time spent in the open and closed arms were recorded using the ANY-maze tracking system (Stoelting Co., Wood Dale, IL). The number of rearings and groomings in the open and closed arms, and the number of head dips in the open arms were also scored by the experimenter. Data were analyzed using two-way (gender×genotype) ANOVA.

### Open-field Assay

Each mouse was placed into the center of a clear Plexiglas chamber (40 cm×40 cm×30 cm) with photo beams to record horizontal and vertical movements of the mouse. Activity was recorded over a 30-min period using a computer-operated VersaMax Animal Activity Monitor System (Acuscan Instruments, Columbus, OH). Testing was performed in the presence of overhead bright lights (∼750 lux of illumination) and white noise (55 dB). Data were collected in 2 min intervals and the following measures were analyzed: total distance traveled (cm), vertical activity, time spent in center zone and the center to total distance ratio. These ratios were calculated by dividing the center distance (distance traveled in the arena center: 22.5 cm×22.5 cm) by total distance traveled. Total distance traveled data in 10 min intervals were analyzed using a three-way (gender×genotype×time) ANOVA with repeated measures. All other data for the 30 min period were analyzed using two-way (gender×genotype) ANOVA.

### Acoustic Startle and Prepulse Inhibition of Acoustic Startle

Prepulse inhibition of the acoustic startle response was measured as described in Paylor and Crawley [27]. Briefly, acoustic startle responses were measured using the SR-Lab startle response system (San Diego Instruments, San Diego, CA). Each mouse was placed in a Plexiglas cylinder within a sound-attenuating chamber and habituated to a 70-dB background white noise for 5 min prior to beginning the test session. Each test session consisted of six blocks, with each block containing eight pseudo-randomized trial types. These include: no stimulus (to measure baseline movement in the cylinder), startle stimulus only (120-dB, 40 ms), and three prepulse stimuli (74, 78, 82-dB; 20 ms) presented either alone or 100 ms before the startle stimulus. The inter-trial intervals ranged from 10 to 20 s. Startle responses, detected as force changes within the Plexiglas cylinder, were recorded every 1 ms during a 65 ms period that followed the onset of either the prepulse during prepulse-alone trials or the startle stimulus. The maximum startle amplitude was used as the dependent variable. Percent PPI of the startle response was calculated for each prepulse as 100– [(startle response to trials with prepulse and startle stimulus trials/startle response to trials with startle stimulus alone)×100].

Acoustic startle response amplitude data were analyzed using two-way (gender×genotype) ANOVA. PPI data were analyzed using a three-way (gender×genotype×prepulse sound level) ANOVA with repeated measures. One mouse did not meet our criterion for minimum startle response to the 120 dB sound stimulus (100) and therefore it was excluded in the analysis.

### Rotarod Test

Motor coordination and skill learning were tested using an accelerating rotarod (UGO Basile, Varese, Italy). Mice were placed on a rotating drum (3 cm in diameter), which accelerated from 4 to 40 rpm over a 5 min period. The time spent walking on top of the rod until the mouse either fell off the rod, or slipped and held onto the rod to ride completely around was recorded. Mice were given four trials on 2 consecutive days with a maximum time of 300 s (5 min) per trial and a 60 min inter-trial rest interval. Rotarod data were analyzed using a three-way (gender×genotype×day) ANOVA with repeated measures.

### Inverted Screen

Each mouse was placed onto a wire grid, and the screen was inverted so that the mouse was hanging upside down from the grid several inches above a plastic covered foam pad. The latency to fall with a cutoff time of 60 sec was measured. Data were analyzed using two-way (gender×genotype) ANOVA.

### Wire-hang and Dowel Assays

For the wire-hang test the mouse was held by the tail and allowed to grasp with its forepaws in the middle of a single 3-mm plastic coated wire suspended 15 inches above a plastic-covered foam pad and released. For the dowel test, the mouse was placed length-wise onto a plastic dowel suspended several inches above a plastic-covered foam pad. Two dowel sizes were used in succession: 3/8-in and ¼-in. Latency to fall was measured with a 60-sec cutoff time for wire-hang, and a 120-sec cutoff time for dowel test. Data were analyzed using two-way (gender×genotype) ANOVA.

### Grip Strength Assay

For this test, each mouse was held by the tail and allowed to grasp the bar of the Chatillon-Ametek grip strength meter with both its forepaws and then the mouse was pulled away from the bar until it released the bar. The test was repeated 5 times for each mouse, and the maximum force generated for each pull was recorded. The highest and lowest scores from individual mouse were removed and the grip strength values were acquired by averaging the scores from three pulls. Data were analyzed with student’s t-test.

### Parallel Rod Footslip Test

The test apparatus was enclosed in a clear Plexiglas chamber (20 cm×20 cm×28 cm); 1.6 mm diameter rods were spaced 6 mm apart and elevated 1 cm above a metal plate. In this assay, each mouse was required to walk and balance on thin (1.6 mm) parallel rods spaced 6 mm apart. Ataxia and locomotor activity were recorded simultaneously for 10 min. Footslips were detected when a paw touched a metal plate below the parallel rod floor, completing a circuit that was scored by the system (Stoelting Co., Wood Dale, IL). Locomotor activity was measured by the ANY-maze tracking system. The number of errors (footslips) per distance traveled (cm) was calculated and analyzed using two-way (gender×genotype) ANOVA.

### Forced Swim Test

Each male mouse was placed for 6 min in a glass beaker (diameter: 13 cm, height: 19 cm) filled with water (height: 14 cm, temperature: 22±1°C). Water was changed between mice. The duration of immobility during the last four minutes of a six minute trial was recorded. Minimal movements made to balance the body and keep the head above the water were scored as immobility. Data were analyzed using Student’s *t*-test.

### Pavlovian Conditioned Fear

Freezing behavior in a conditioned fear paradigm was measured as described previously [28]. The test chamber (26 cm×22 cm×18 cm high) had clear Plexiglas sides and a grid floor bottom that was used to deliver a mild foot shock. The chamber was placed inside a sound-attenuation chamber (Med Associates, St. Albans, VT) that had a window through which mice could be observed without disturbance. On the training day, mice were placed into the test chamber (house lights ON) and allowed to explore for 2 min. The conditioned stimulus (CS, a 80 dB white noise) was presented for 30 s and followed immediately by a mild foot shock (2s, 0.7 mA) that served as the unconditioned stimulus (US). Two minutes later, a second CS-US pairing was presented. The FreezeFrame2 monitor system (Actimetrics, Wilmette, IL) was used to control the timing of CS and US presentations and measure freezing behavior. In the present study, all of the mice responded to the foot shock.

Mice were tested for contextual and cued fear conditioning 24 h after conditioning. For the context test, mice were placed back into the original test chamber for 5 min and freezing behavior was recorded. One to two hours later, mice were tested for responses to the auditory CS in a new environment. For the CS test, white Plexiglas inserts were placed on the sides and floor of the chamber to alter the shape, texture and color of the chamber. Vanilla extract was placed in the chamber behind the insert to alter the odor. Transfer cages were altered (no bedding) and red house lights replaced the normal white house lights. Mice were placed into this new chamber and freezing was recorded for 3 min during this ‘pre-CS’ phase. The auditory CS was then presented for another 3 min and freezing was recorded. Data for the CS test were calculated as the percent freezing during the CS *minus* percent freezing in the pre-CS phase. Data were analyzed using two-way (gender×genotype) ANOVA for the total duration.

### Hot-plate Test

Mice were tested for analgesia-related responses using a hot-plate apparatus (Columbus Instruments, Columbus, OH). The hot-plate was enclosed in a clear Plexiglas chamber and preheated to 55±0.1°C. Each mouse was placed gently onto the hot-plate and the time to initiate a hind limb response was recorded. The same mice were tested on hot-plate preheated to 50±0.1°C on a separate day. Typical nociceptive responses are licking or shaking the hindpaw, or jumping. Mice were immediately removed after showing a response, with a cut-off time of 45 sec. Data were analyzed using two-way (gender×genotype) ANOVA.

### Statistical Analyses

Statistical analysis was conducted using SPSS (SPSS, Chicago, IL, USA), Prism 3.0 (Graph-Pad Software) or SigmaPlot (Systat Software Inc., San Jose, CA). All data were analyzed using two-way ANOVA (gender×genotype), three-way ANOVA with repeated measures (gender×genotype×time), or student’s t-test (genotype) if only male mice were tested. All data are presented as mean ± SEM. The level of significance was set at *P*≤0.05.

## Results

### General Characteristics of GPR55 KO Mice

**Figure 1:**
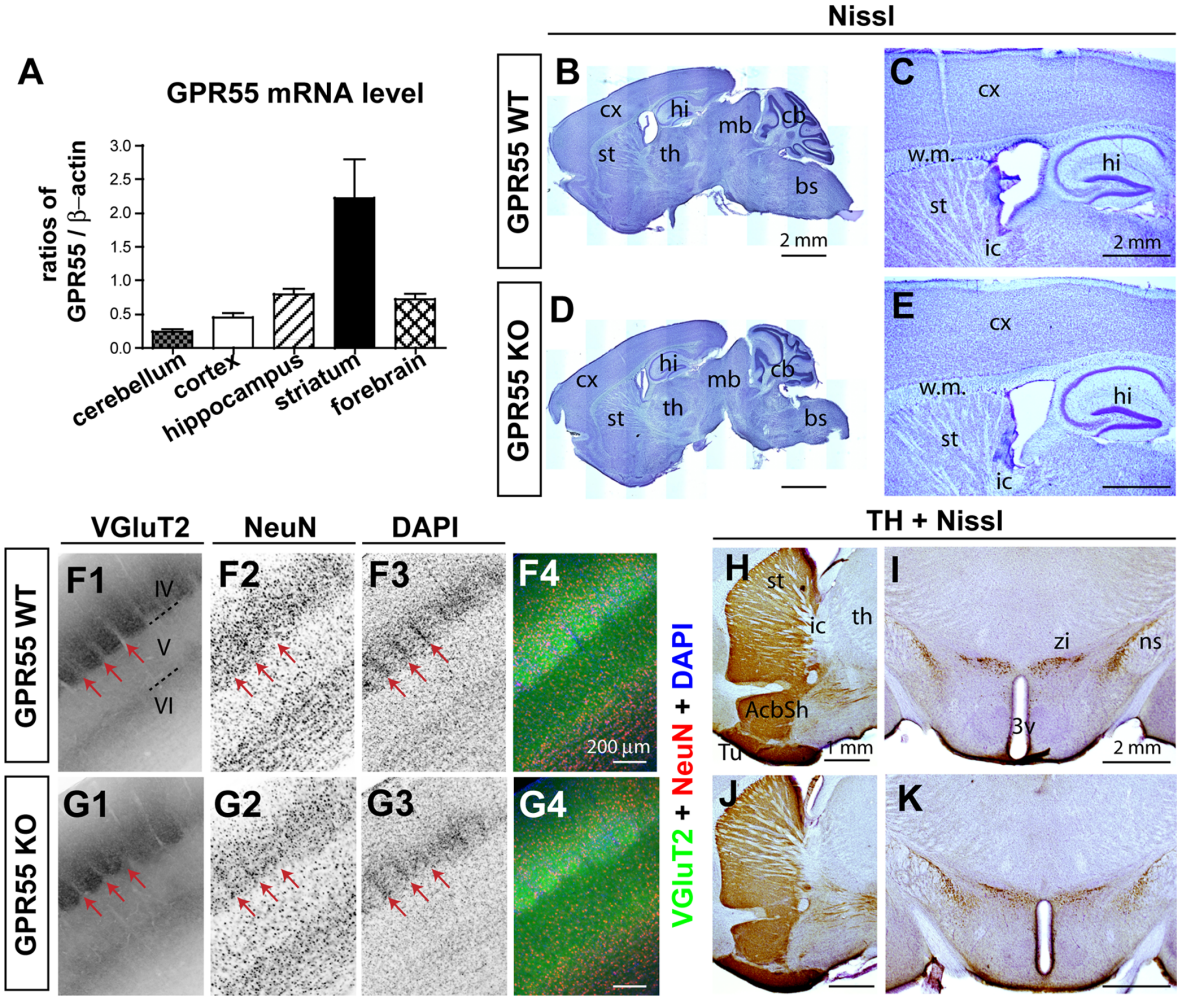
GPR55 is expressed in mouse brains but GPR55 deletion does not alter gross brain structures. (**A**) Expression levels of GPR55 mRNA in different brain regions. Data are presented as mean ± SEM. (**B–E**) Nissl staining show that brain structures in GPR55 KO mice appear similar to WT littermates. (**B, D**) Montages of low magnification images show an overview of the brain in sagittal planes. (**C**,**E**) Higher magnification Nissl images show the cortex, hippocampus, and striatum in WT (**C**) and GPR55 KO (**E**) brains. (**F**–**G**) Whisker-related barrel patterns in the primary somatosensory cortex of GPR55 KO mice appear similar to WT littermates (coronal planes). The barrel pattern is revealed by the TCA marker VGluT2 (**F1**, **G1**) and the neuronal marker NeuN (**F2**, **G2**) co-staining. Sections were counterstained with DAPI (**F3**, **G3**), and merged images are shown in **F4**, **G4**. Arrows indicate the location of barrel walls. (**H**–**K**) Pattern of TH-immunopositivity in the striatum (sagittal planes; **H**, **J**) and midbrain (coronal planes; **I, K**). Abbreviations: IV–VI, cortical layer IV–VI; 3v, the third ventricle; AcbSh, nucleus accumbens shell; bs, brain stem; cb, cerebellum; cx, cortex; hi, hippocampus; ic, internal capsule; mb, midbrain; ns, nigrostriatal bundle; st, striatum; TCA, thalamocortical axon; th, thalamus; Tu, olfactory tubercle; zi, zona incerta; w.m., white matter.

In human brain, Northern blotting detected GPR55 mRNA in the caudate and putamen, but not in the frontal cortex, hippocampus, thalamus, pons, or cerebellum [29]. Using quantitative PCR we found that GPR55 mRNA was expressed in mouse brain in the following order: striatum>hippocampus>forebrain>cortex>cerebellum (**Fig. 1A**). The abundance of various components of the endocannabinoid system in GPR55 KO hippocampus was also examined. We found no alterations in the levels of the 2-AG degrading enzymes alpha/beta-hydrolase domain containing 6 enzyme (ABDH6) and monoacyl glycerol lipase (MGL), the 2-AG synthesizing enzymes diacylglycerol lipase alpha and beta (DGLα and DGLβ), nor the AEA degrading enzyme fatty acid amide hydrolase (FAAH) (**Table 1**).

**Table 1:** Characterization of endocannabinoid system in GPR55 KO mouse hippocampus.

qPCR-ECS components	WT	KO
ABDH6	1.0±0.17 (4)	1.3±0.06 (6)
DGLα	1.0±0.01 (4)	0.9±0.09 (6)
DGLβ	1.0±0.11 (5)	1.0±0.11 (7)
FAAH	1.0±0.14 (4)	0.9±0.09 (6)
MGL	1.0±0.13 (9)	1.2±0.12 (10)
**Lipid**	**WT**	**KO**
2-AG (nmoles/gram)	17.2±2.0 (6)	16.7±2.4 (6)
2-LG (nmols/gram)	0.53±0.05 (6)	0.37±0.01 (5)
AEA (pmoles/gram)	4.92±0.54 (6)	4.49±0.66 (6)
LEA (pmoles/gram)	8.37±1.16 (6)	7.35±0.86 (6)
OEA (pmoles/gram)	27.82±4.9 (6)	21.4±1.1 (6)
PEA (pmoles/gram)	7.55±1.5 (6)	6.62±.13 (6)
NAGly (pmoles/gram)	32.1±2.81 (6)	35.7±2.3 (6)
PalGly (pmoles/gram)	25.3±4.08 (6)	28.1±4.21 (6)
AraSer (pmoles/gram)	57.6±4.93 (6)	63.3±8.74 (6)
OlSer (nmoles/gram)	0.22±0.03 (6)	0.25±0.06 (6)
PGE2 (nmoles/gram)	0.83±0.11 (6)	0.87±0.10 (6)
PGF2α (nmoles/gram)	1.99±0.13 (6)	1.94±0.18 (6)

Values are listed as mean ± SEM (number of animals analyzed). Abbreviations: ABHD6, alpha/beta-hydrolase domain containing 6 enzyme; MGL, monoacyl glycerol lipase; DGLα, diacylglycerol lipase alpha; DGLβ, diacylglycerol lipase beta; and FAAH, fatty acid amide hydrolase. AEA, arachidonoylethanolamide; OEA, oleoylethanolamide; LEA, linoleoylethanolamide; PEA, palmitoylethanolamine; 2-AG, 2-arachidonoyl glycerol; 2-LG, 2-linoleoyl glycerol; NAGly, *N*-arachidonoyl glycine; PalGly, *N*-palmitoyl glycine; AraSer, N-arachidonoyl serine; OlSer, N-oleoyl serine; PGE2, prostaglandin E2; PGF2α, prostaglandin F2α.

Anandamide (N-arachidonoyl ethanolamide; AEA) and 2-arachidonoyl glycerol (2-AG) are two major endogenous ligands of cannabinoid receptors [30]. N-linoleoyl ethanolamide (LEA), N-palmitoyl ethanolamide (PEA), oleoylethanolamide (OEA), and 2-linoleoylglycerol (2-LG) are endogenous fatty acid derivatives that structurally resemble the endocannabinoids. 2-LG has been shown to potentiate the action of endocannabinoids [31], and LEA, PEA, and OEA have been shown to exert cannabimimetic activity [32]. N-arachidonoyl glycine (NAGly) is an endogenous AEA metabolite and activates GPR18 [33], [34] and N-palmitoyl glycine (PalGly) is an endogenous analog that increases calcium in DRG neurons [35]. N-arachidonoyl serine (AraSer) and N-oleoyl serine (OlSer) are endongenous lipids with a variety of biological activity through as yet unknown receptors [36], [37]. Prostaglandin E2 (PGE2) and prostaglandin F2α (PGF2α) were recently shown to be metabolites of 2-AG in the brain and their activity directly related to endogenous cannabinoid function [38]. To explore whether deleting GPR55 affects the levels of endogenous cannabinoids and related lipids, lipid extracts of hippocampi of GPR55 KO mice and their wild-type (WT) littermates were prepared for mass spectrometric analysis. Similar levels of the endocannabinoids AEA and 2-AG, and the levels of endocannabinoid related compounds OEA, LEA, PEA, NAGly, PalGly, AraSer, OlSer, PGE2, PGF2α, and 2-LG (**Table 1**) were found in GPR55 KO and WT hippocampi. Taken together with the qPCR data, we found that GPR55 deletion did not change either components of the hippocampal endocannabinoid system at the transcriptional level or eCB homeostasis.

GPR55 KO mice are fertile and show no obvious alteration in physical characteristics (appearance of fur and whiskers). There were no significant differences in body weight between male or female GPR55 KO and their WT littermates (genotype: F_1, 59_ = 0.105, p = 0.747, gender: F_1, 59_ = 144.210, p<0.001; **Table 2**). The ability of GPR55 to stimulate Rho-mediated pathways and increase intracellular calcium suggests that it may play a role in cell migration and neurite outgrowth [9]. To survey brain morphology at the gross level, NISSL staining was conducted with six-week old GPR55 KO (n = 3) and their littermate control mice (n = 3). No obvious alteration in brain morphology was detected in GPR55 KO mice (**Fig. 1B–E**). Immunostaining was used to probe specific functional circuits. Double labeling with a thalamocortical axon (TCA) marker, vesicular glutamate receptor 2 (VGluT2), and the neuronal marker NeuN revealed distinctive whisker-related patterns in the primary somatosensory cortex (**Fig. 1F,G**). Segregated TCA clusters relaying sensory information from single whiskers flanked by layer IV neurons were equally evident in the S1 cortex of both genotypes. Tyrosine hydroxylase (TH) is the rate-limiting enzyme for catecholamine synthesis [39] and is a useful marker for dopaminergic and noradrenergic circuits [40]. TH immunohistochemistry reveals identical patterns of intense fibrous TH immunoreactivity throughout the striatum and substantia nigra in both GPR55 KO and control brains (**Fig. 1H–K**). These data suggest that deleting GPR55 function does not grossly affect the development of the circuits examined.

**Table 2:** Summary of behavioral data.

Behavioral paradigm	Measurement	Male		Female		Genotype effect	Gender effect
		WT	KO	WT	KO	p value	p value
	body weight (g)	27.8±0.8 (12)	27.2±0.4 (19)	20.8±0.4 (15)	21.7±0.5 (17)	0.747	<0.001 *
**Hotplate**	time to hindlimb response at 50°C (s)	18.5±2.7 (8)	16.1±1.2 (13)	13.8±2.5 (8)	19.7±1.6 (10)	0.386	0.764
	time to hindlimb response at 55°C (s)	14.5±2.2 (8)	14.0±1.2 (13)	16.6±1.6 (8)	10.3±1.1 (10)	0.031 *	0.607
**Elevated Plus Maze**	time spent in open arm (s)	154.6±27.1 (9)	173.8±17.2 (17)	181.7±27.3 (12)	189.7±17.1 (17)	0.535	0.329
	open arm entries #	16.0±1.0 (9)	15.5±1.0 (17)	16.8±1.9 (12)	18.9±1.6 (17)	0.608	0.187
	head-dips #	27.4±3.9 (9)	26.6±2.3 (17)	25.7±2.9 (12)	31.5±3.0 (17)	0.412	0.614
	rearings #	32.8±5.4 (9)	26.2±2.2 (17)	28.3±2.9 (12)	26.4±1.9 (17)	0.154	0.465
	groomings #	6.0±1.3 (9)	4.3±0.6 (17)	4.3±0.9 (12)	5.4±0.7 (17)	0.705	0.740
	distance traveled in close arm (m)	6.3±0.66 (9)	5.8±0.41 (17)	6.5±0.60 (12)	5.9±0.33 (17)	0.35	0.929
	line-crossings #	82.3±3.6 (9)	76.1±3.9 (17)	88.9±8.7 (12)	83.6±4.7 (17)	0.315	0.222
**Open Field**	total distance traveled (cm)	3651. ±274.1 (12)	3643.6±247.2 (19)	4254.9±309.5 (15)	3373.2±210.0 (17)	0.089	0.553
	vertical activity (# beam breaks)	357.5±46.5 (12)	357.7±38.9 (19)	229.1±23.4 (15)	203.6±30.9 (17)	0.728	<0.001 *
	time in center zone (s)	306.9±37.2 (12)	349.3±40.4 (19)	292.5±30.6 (15)	263.9±35.2 (17)	0.854	0.189
	ratios of center to total distance	0.26±0.02(12)	0.26±0.02 (19)	0.26±0.02 (15)	0.23±0.02 (17)	0.438	0.529
**Forced Swim Test**	% immobility	55.3±6.9 (10)	59.5±6.0 (13)	N.A.	N.A.	0.651	N.A.
**Inverted Screen**	time to fall (s)	50.9±6.06 (9)	45.7±5.5(15)	60.0±0.0 (12)	60.0±0.0 (16)	0.346	0.021 *
**Wire-hang**	time to fall (s)	38.9±8.1 (8)	40.8±8.7 (6)	54.5±3.28 (11)	45.1±7.4 (8)	0.590	0.152
**Grip Strength**	Maximum force (A.U.)	0.11±0.029 (11)	0.11±0.005 (11)	N.A.	N.A.	0.564	N.A.
**Dowel Test**	time to fall (3/8 inch rod) (s)	31.5±6.8 (8)	28.0±11.1(6)	42.2±9.7 (11)	45.3±11.4 (8)	0.324	0.225
	time to fall (1/4 inch rod) (s)	26.0±5.9 (8)	38.7±9.5 (6)	41.4±10.2 (11)	51.5±15.5 (8)	0.983	0.182
**Footslip Assay**	normalized errors (# errors/distance)	22.2±3.4 (8)	53.4±12.1 (6)	23.7±4.2 (11)	43.1±14.9 (8)	0.01 *	0.638
	distance traveled (m)	10.8±1.3 (8)	8.5±1.0 (6)	12.2±0.7 (11)	10.8±1.0 (8)	0.083	0.082
**Prepulse Inhibition**	% PPI at 74 dB prepulse	5.8±6.9 (12)	7.9±4.4 (19)	12.5±7.1 (15)	­0.1±4.7 (17)	N.A.	N.A.
	% PPI at 78 dB prepulse	13.4±6.8 (12)	27.9±3.9 (19)	21.6±5.8 (15)	23.5±5.0 (17)	N.A.	N.A.
	% PPI at 82 dB prepulse	29.1±8.1 (12)	38.8±4.6 (19)	36.0±5.1 (15)	43.1±5.1 (17)	N.A.	N.A.
	normalized startle response (A.U./gbody weight)	41.3±4.6 (12)	33.3±4.1 (19)	35.6±2.5 (15)	18.3±2.3 (17)	0.001 *	0.005 *
**Fear Conditioning**	% freezing in context test	33.1±5.6 (12)	28.7±3.8 (19)	31.6±3.71 (15)	35.3±3.2 (17)	0.931	0.533
	% freezing in cued test	63.8±3.2 (12)	65.7±2.6 (19)	66.1±3.9 (15)	68.8±2.7 (17)	0.194	0.252

Values are listed as mean ± SEM (number of animals analyzed). Abbreviations: A.U., arbitrary unit;

# number;

* statistically significant difference; N.A., not analyzed.

### Normal Synaptic Function and Plasticity in GPR55 KO Hippocampus

**Figure 2:**
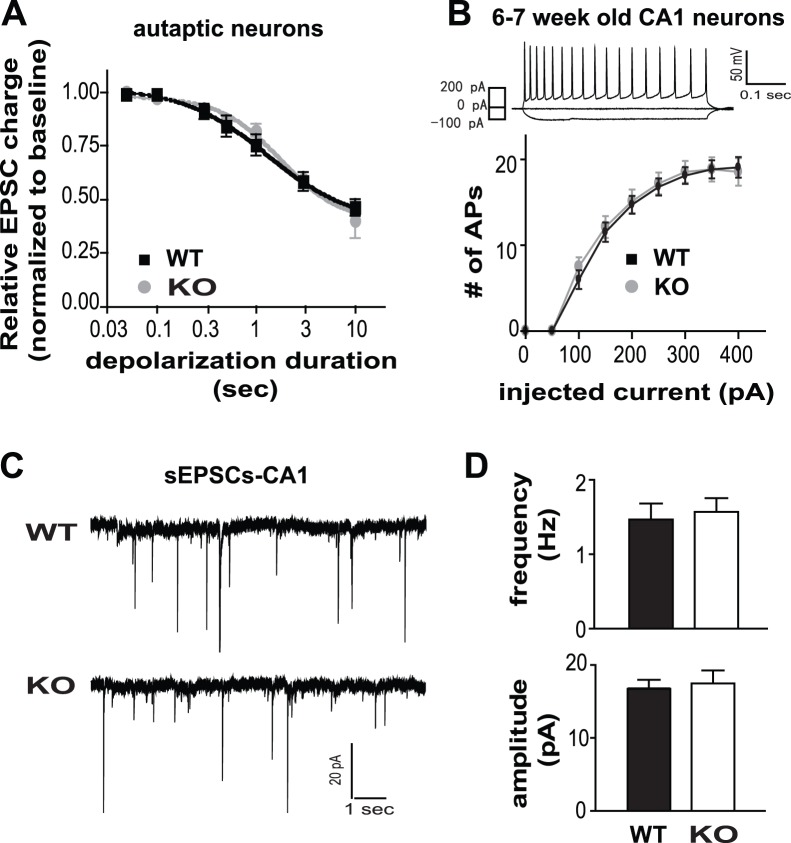
GPR55 deletion does not affect short-term endocannabinoid-mediated synaptic plasticity or CA1 pyramidal neuron excitability. (**A**) Depolarization-induced suppression of excitability (DSE) sensitivity and strength were assessed in hippocampal neurons cultured from GPR55 KO or WT mice. (**B**) Normal intrinsic excitability in GPR55 KO CA1 pyramidal neurons. Top panel: Example recordings show typical adapting synaptic responses of a WT CA1 neuron triggered by injecting current through the recording pipette. Bottom panel: Summary of the input-output relationships between the number of evoked APs and the amount of injected current. (**C**) Representative traces of spontaneous EPSCs (sEPSCs) recorded in CA1 pyramidal neurons from GPR55 KO and WT littermate mice. (**D**) Summaries for sEPSC frequencies and amplitudes.

Depolarization-induced suppression of excitation (DSE) is a well-characterized form of endocannabinoid (eCB)-mediated synaptic plasticity. DSE sensitivity and strength can be quantified by constructing a “dose-response” curve where the suppression of excitatory postsynaptic potentials to progressively longer depolarizations is determined. To examine whether GPR55 deletion affects eCB-dependent DSE, autaptic culture neurons were prepared from GPR55 KO and control pups. The DSE dose response curves were very similar in neurons cultured from GPR55 KO or WT mice (**Fig. 2A**). These data demonstrate that GPR55 deletion does not affect this form of eCB signaling in cultured autaptic hippocampal neurons.

GPR55 activation inhibits the potassium M-current in HEK293 cells expressing GPR55 [9]. This finding suggests that GPR55 may enhance neuronal excitability. To probe whether excitability or synaptic connections were altered in GPR55 KO neurons, whole-cell recordings were conducted with CA1 pyramidal neurons in four pairs of 6–7 week old GPR55 KO mice and their WT littermates. Whole-cell current-clamp recordings were used to examine the intrinsic membrane properties of CA1 pyramidal neurons. Injecting somatic depolarizing current pulses evoked a train of action potentials (APs) typical of CA1 pyramidal neurons (**Fig. 2B**). Both the intrinsic membrane properties (**Table 3**) and the excitability, defined by the number of APs triggered by increasing amounts of current, were similar between control (n = 22) and GPR55 KO CA1 pyramidal neurons (n = 16) (**Fig. 2B**). Next, CA1 neurons were recorded in voltage-clamp mode to detect spontaneous events to evaluate the synaptic connections onto CA1 neurons. No differences in the frequency or amplitude of spontaneous EPSCs (sEPSCs) were found between these two groups (sEPSC amplitude: WT, 16.72±1.26 pA, n = 26; KO, 17.48±1.78 pA, n = 22; sEPSC frequency: WT, 1.47±0.22 Hz, n = 26; KO, 1.57±0.18 Hz, n = 22) (**Fig. 2C–D**). These results show that the absence of GPR55 does not affect the intrinsic excitability or connectivity of CA1 pyramidal neurons.

**Table 3:** Summary of intrinsic membrane properties of hippocampal CA1 neurons.

Membrane Properties	WT (n = 15)	KO (n = 15)	p value
**Ra (MΩ)**	22.4±1.9	24.0±2.7	0.6343
**Rm (MΩ)**	163.4±17.4	167.8±22.4	0.8779
**Tau (µs)**	825.2±52.6	825.5±95.2	0.998
**Cm (pF)**	43.6±2.9	44.9±5.1	0.8206
**Vrest (mV)**	­62.8±0.5	­62.3±0.4	0.4869

Values are listed as mean ± SEM and analyzed with unpaired Student’s t test (two-tailed). Abbreviations: Vrest, resting potential; Ra, access resistance; Rm, membrane resistance; Cm, membrane capacitance; Tau, time constant.

**Figure 3:**
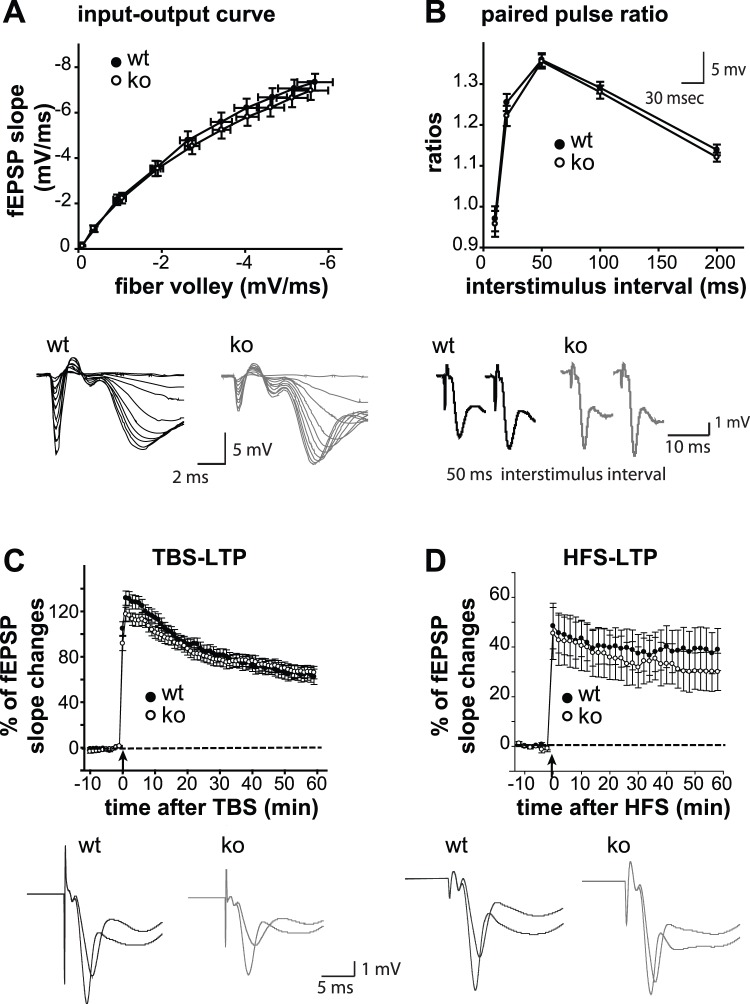
Normal synaptic transmission and plasticity in GPR55 KO hippocampal CA1 pyramidal neurons. (**A**) Input-output relationships in the Schaffer collateral pathway are identical between GPR55 KO and WT mice. (**B**) Paired-pulse ratios are similar between GPR55 KO and WT slices (PP10 ratios: WT = 0.97±0.03, KO = 0.96±0.03; PP20 ratios: WT = 1.25±0.02, KO = 1.22±0.03; PP50 ratios: WT = 1.36±0.02, KO = 1.36±0.02; PP100 ratios: WT = 1.29±0.01, KO = 1.28±0.02; PP 200 ratios ISI: WT = 1.14±0.01, KO = 1.12±0.01). (**C**–**D**) Summaries show long-term potentiation (LTP) induced by TBS (**C**), or HFS protocol (**D**) in both WT and GPR55 KO Schaffer collateral pathways.

To further explore the potential contribution of GPR55 in regulating synaptic function and plasticity, field potential recordings were conducted in the stratum radiatum of CA1 with stimulation of the Schaffer collateral pathway in acute hippocampal slices prepared from GPR55 KO and their WT littermate mice at 3 months of age. We first analyzed general synaptic transmission by examining the input-output relationship of synaptic responses in the CA1 area over a range of stimulus intensities (**Fig. 3A** **;** WT, 28 recordings from six animals; KO, 31 recordings from seven animals). Similar input–output relationships were found when comparing fEPSP slope with fiber volley amplitude between GPR55 knockout mice and control mice (fiber volley: F_1, 57_ = 0.001, p = 0.978; fEPSP: F_1, 57_ = 0.274, p = 0.603; two-way ANOVA with repeated measure (genotype×stimulus intensity). These data indicate that synaptic transmission is grossly normal in the GPR55 KO Schaffer collateral pathway.

Next, short-term plasticity was evaluated with paired stimuli separated by a 10, 20, 50, 100, or 200 msec inter-stimulus intervals (**Fig. 3B**). Normal paired-pulse facilitation was observed in GPR55 KO synapses when inter-stimulus intervals were greater than 10 msec (F_1, 57_ = 0.479, p = 0.492; two-way ANOVA with repeated measure (genotype×stimulus interval)). To examine whether long-term synaptic plasticity was defective in GPR55 KO mice, both theta-burst stimulation (TBS; WT, 27 recordings from six animals; KO, 28 recordings from seven animals) and high frequency stimulation protocols (HFS; WT, 17 recordings from six animals; KO, 15 recordings from six animals) were used to induce the formation of long-term potentiation (LTP) at Schaffer collateral synapses. Strong and persistent LTP was induced with either TBS or HFS in GPR55 KO CA1 (**Fig. 3C,D**). No difference in the degree of potentiation was found between the two genotypes (TBS-LTP, t_53_ = 0.558, p = 0.579; C; HFS-LTP, t_30_ = 0.274, p = 0.786). Taken together, deleting GPR55 function has no impact on the types of short-term and long-term plasticity examined.

### Elucidating the Role of GPR55 in CNS with a Panel of Behavioral Assays

To address the behavioral effects of GPR55 deletion, we subjected 2–5 month old male and female GPR55 KO mice and their WT littermates to a comprehensive battery of behavioral tests.

### Sensory Function

Staton et al. [11] reported that female GPR55 KO mice showed a reduction in withdrawal latency in the hot-plate test, an acute pain test for thermal nociception deficits, when tested at 50°C, but not at higher temperatures (52.5°C, 55°C). To see whether our GPR55 KO mice show similar deficits as the earlier strain, we tested these mice on the hot-plate assay. In contrast to the previous report, there was no significant genotype nor gender effect on time to hindlimb response at the lowest temperature tested (50°C; genotype: F_1, 35_ = 0.772, p = 0.386, gender: F_1, 35_ = 0.092, p = 0.764). However, there was a significant genotype effect but no gender effect on time to hindlimb response at higher temperature (55°C; genotype: F_1, 35_ = 5.028, p = 0.031, gender: F_1, 35_ = 0.269, p = 0.607) (**Table 2**). There was significant genotype×gender interaction effects at 50°C (F_1, 35_ = 4.564, p = 0.04) but no significant interaction effects at 55°C (F_1, 35_ = 3.709, p = 0.062). To compare to the previous report, the data were analyzed separately for male and female mice. Similar to previous report, it was the female GPR55 KO mice that showed a significant reduction in withdrawal latency at 55°C when compared to WT littermates (female mice: t_16_ = 3.363, p = 0.004; male mice: t_19_ = 0.208, p = 0.838). Thus, loss of GPR55 perturbs thermal nociception.

### Normal Baseline Anxiety Level and Spontaneous Activity in GPR55 KO Mice

To explore the possible role of GPR55 in regulating anxiety levels in mice, we performed both elevated plus maze and open field tests. The elevated plus maze assay is a well-validated behavioral test to measure anxiety in rodents [26] There was no overall difference between the genotypes or genders in the amount of time spent in open arms (**Table 2**; genotype: F_1, 54_ = 0.391, p = 0.535; gender F_1, 54_ = 0.973, p = 0.329) or in the number of entries to open arms (**Table 2**; genotype F_1, 54_ = 0.266, p = 0.608; gender F_1, 54_ = 1.791, p = 0.187). No significant gender×genotype interaction effect was observed in either parameter. In addition, no overall differences between the genotypes and genders were found in other measures of anxiogenic- or anxiolytic-like behaviors monitored, such as number of head-dips in the open arms (exploratory behavior), number of rearings, and number of groomings (**Table 2**). The activity levels of GPR55 KO and WT littermates in the elevated plus maze, indicated by distance traveled in closed arms, were not significantly different (**Table 2**; genotype: F_1,54_ = 0.888, p = 0.35, gender: F_1,54_ = 0.008, p = 0.929). There was no overall difference in the total number of entries between GPR55 KO and WT mice either, another indication that the absence of GPR55 activity has no impact on spontaneous activity in the elevated plus maze (**Table 2**; genotype: F_1,54_ = 1.032, p = 0.315, gender: F_1,54_ = 1.529, p = 0.222).

**Figure 4:**
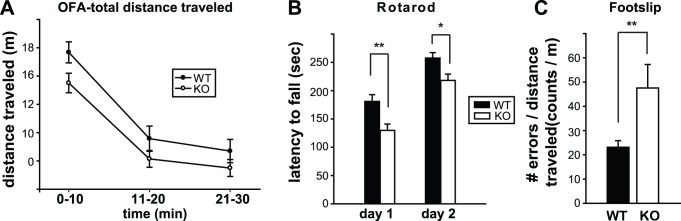
GPR55 KO mice have impaired motor coordination. (**A**) Summary for the novelty-induced locomotion measured in an open field assay. Horizontal activities were measured as total distance traveled. (**B**) Motor skill learning and coordination was assessed in the accelerating rotarod test. The time spent walking on top of the rotarod (latency to fall) for two consecutive days is shown for GPR55 KO mice and their WT littermates. There was a significant difference between genotypes (Day 1: t_ 61_ = 3.061, p = 0.003; Day 2: t_61_ = 2.569, p = 0.013; Student’s t-test), with GPR55 KO mice performing worse than their littermate controls for both days. (**C**) GPR55 KO mice made significantly more mistakes (footslips) while walking on parallel rods compared to littermate controls. *p<0.05; **p≤0.01.

The open field paradigm allows for simultaneous assessment of novelty-induced exploratory activity and anxiety levels [26]. To explore reactivity to novel environment and within-session habituation during the 30 min test time, the data were analyzed in 3 time brackets using two-way ANOVA with repeated measures (genotype×interval) (**Fig. 4A**). Gender data were combined since there was no genotype×gender interaction effect (F_1, 59_ = 2.645, p = 0.109). GPR55 KO mice showed a trend towards decreased horizontal activity throughout the 30 min time period, but this was not statistically significant (F_1, 61_ = 3.318, p = 0.073). Both GPR55 KO mice and WT littermates decreased their exploratory activities as testing progressed (time in 3 blocks, F_1.56, 95.34_ = 128.418, p<0.001), indicating habituation to the novel environment. There was no genotype×interval interaction effects (F_1.56, 95.34_ = 0.644, p = 0.491). For vertical activity, there was a significant gender difference but the activity levels were comparable between GPR55 KO and littermate control mice in both male or female mice (genotype: F_1,59_ = 0.122, p = 0.728, gender: F_1,59_ = 15.311, p<0.001). There was no significant gender×genotype interaction effect (F_1,59_ = 0.127, p = 0.723).

The ratio of center distance to total distance traveled (to normalize for activity levels) and time spent in the center zone measured in the open field assay provide measures of anxiety-related responses to a bright and open arena [41], [42]. GPR55 KO mice were indistinguishable from littermate controls in both center distance ratio (**Table 2**; genotype: F_1,59_ = 0.61, p = 0.438, gender: F_1,59_ = 0.401, p = 0.529) and time spent in center zone (genotype: F_1,59_ = 0.034, p = 0.854, gender: F_1,59_ = 1.767, p = 0.189). Integrating the results of these studies, GPR55 KO mice showed essentially normal baseline anxiety levels and slightly reduced reactivity to a novel environment, but habituated normally.

Cannabinoids are also known to influence depressive behaviors [43]. Depressive behavior was assessed in GPR55 KO and littermate control mice using the Forced Swim Test, one of the most widely used tools for screening antidepressants [44]. There was no significant difference (**Table 2**; t_21_ = 0.459, p = 0.651) in immobility time between GPR55 KO (n = 13, 59.5±5.98 seconds) and WT (n = 10, 55.3±6.93 seconds). These data suggest that GPR55 KO mice do not exhibit overt depressive behaviors.

### Impairment of Motor Coordination in GPR55 KO Mice

GPR55 is expressed in the basal ganglia and cerebellum (**Fig. 1**), important areas for motor function [45], [46], [47]. To explore the potential role of GPR55 in motor coordination/control and balance, we tested GPR55 KO and WT mice on the accelerating rotarod (**Fig. 4B**). The mice were subjected to 4 trials per day for two consecutive days, and the averaged data for each day were analyzed using three-way ANOVA (gender×genotype×day) with repeated measures. There were significant overall differences between the genotypes (F_1, 59_ = 9.292, p = 0.003) and genders (F_1, 59_ = 4.896, p = 0.031), but no genotype×gender interaction effects (F_1, 59_ = 0.475, p = 0.494), suggesting that GPR55 KO mice performed significantly worse than littermate controls on the rotarod. There was an overall significant time effect (F_1, 59_ = 143.785, p<0.001), but no significant gender×time (F_1, 59_ = 1.152, p = 0.287), genotype×time (F_1, 59_ = 0.625, p = 0.423), or gender×genotype×time (F_1, 59_ = 2.111, p = 0.152) interaction effects. This suggested that both male and female GPR55 KO and littermate control mice improved significantly during training, indicating that GPR55 KO mice were able to learn as the time they stayed on a rotating rod significantly increased over two consecutive days of testing. In summary, GPR55 KO mice exhibited significant motor coordination deficits but their motor performance improved with repeated training.

To further explore this issue, we examined whether GPR55 KO mice fall more rapidly from the accelerating rotarod due to reduced grip strength by conducting inverted screen, wire hang and active grip strength tests. In the inverted screen test, each mouse was required to hang onto a grid while being inverted for 60 sec. No significant differences were found between GPR55 KO and WT littermate mice in grip strength (Table 2; t(20) = 0.59, p = 0.564). In the wire-hang test, each mouse was required to grasp onto a 3-mm suspended wire for 60 sec. The results of the wire-hang test showed no overall gender or genotype effects, (**Table 2**; genotype: F_1, 49_ = 0.927, p = 0.59, gender: F_1, 49_ = 2.162, p = 0.152). In the grip strength test, each mouse was allowed to grasp onto a bar and the maximum force generated while pulling the mouse away from the bar was measured. There was no significant difference between GPR55 KO mice and littermate controls (Table 2; t_12_ =  −0.112, p = 0.913). The results of these assays suggest that GPR55 KO mice have normal grip strength. We also tested the mice on two different dowel assays to assess their ability to remain on a narrow beam. Two beam sizes (3/8 and ¼ inch rods) were used. No significant differences were found between GPR55 KO and WT littermates (**Table 2**).

Next, we challenged GPR55 KO mice with the parallel rod footslip assay to further examine their motor coordination. This test allows the simultaneous measurement of ataxia and locomotor activity [48]. GPR55 KO mice performed significantly worse than WT littermates in terms of the number of footslips/errors per cm traveled on thin parallel rods (genotype: F_1, 29_ = 7.577, p = 0.01, gender: F_1, 29_ = 0.227, p = 0.638). There was no genotype×gender effect on the number of footslips per cm traveled (F_1, 29_ = 0.409, p = 0.528). For clarity, male and female data were combined and shown in **Fig.** **4C**. GPR55 KO mice made significantly more errors than WT littermates (t_31_ = −2.75, p = 0.01). Taken together, these results show that GPR55 KO mice exhibit an ataxia-like phenotype due to motor coordination deficits.

### Normal Sensory-motor Gating in GPR55 KO Mice

**Figure 5:**
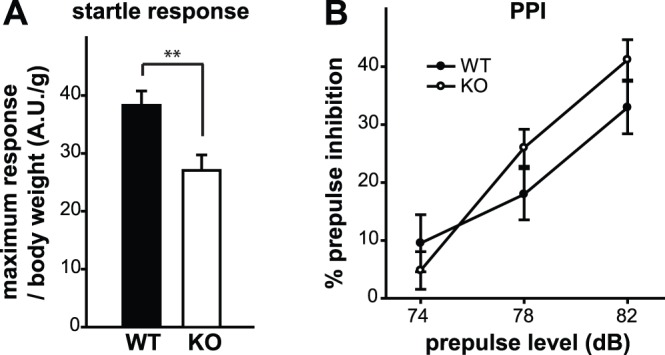
Normal sensory-motor gating in GPR55 KO mice. (**A**, **B**) Sensory-motor gating was measured by prepulse inhibition (PPI) of the acoustic startle response. (**A**) Maximum startle response to a 120 dB white noise sound burst, normalized to body weight, is shown. GPR55 KO mice showed a significantly reduced startle response compared to littermate controls. (**B**) Inhibition of the acoustic startle response was determined with three prepulse levels (74, 78 and 82 dB). GPR55 KO mice showed similar PPI compared with WT littermates at all prepulse levels tested. **p≤0.01.

The prepulse inhibition of acoustic startle reflex (PPI) is the phenomenon in which a weak non-startling sound suppresses the startle response to a strong acoustic startle stimulus presented immediately after the pre-stimulus. As the prepulse level increases, there will be greater suppression of the startle response. PPI provides an operational measure of sensory-motor gating processes in humans and mice (reviewed in [49]). Overall significant effects of genotype and gender were observed in the maximum response (**Table 2**; genotype: F_1, 59_ = 12.942, p = 0.001, gender: F_1, 59_ = 8.706, p = 0.005). There was no genotype×gender interaction effect (F_1, 59_ = 1.801, p = 0.185). For clarity, male and female data were combined and shown in **Fig.** **5A** (t_60_ = 3.0, p = 0.004)**.** For PPI, there was a main effect of prepulse level (F_2, 118_ = 65.234, p<0.001) as expected. Thus, as the prepulse level increases, there is greater suppression of the startle response. We found no significant difference in the percentages of PPI between GPR55 KO mice and WT littermates (genotype: F_1, 59_ = 0.67, p = 0.416, gender: F_1, 59_ = 0.246, p = 0.622; **Fig. 5B**). There was no gender×prepulse level (F_2, 118_ = 0.704, p = 0.497) or genotype×gender×prepulse level interaction effects (F_2, 118_ = 0.731, p = 0.483). In summary, GPR55 KO mice exhibit normal sensory-motor integration.

### Normal Fear-conditioning in GPR55 KO Mice

To explore the role of GPR55 in learning, we employed the conditioned fear paradigm associating contextual or auditory cues with mild foot shock. It is believed that contextual and auditory-cue conditioned fear are mediated by hippocampal and amygdala-dependent processes, respectively [50]. Similar levels of freezing for both contextual and auditory cue-based fear tests performed 24 h after training were found for both GPR55 KO mice and their littermate controls (**Table 2**). In the context test, mice were placed back into the chamber in which they had received conditioning and their freezing levels were measured for 5 min. There were no significant genotype, gender or interaction effects found in the context test (genotype: F_1, 59_ = 0.008, p = 0.931, gender: F_1, 59_ = 0.392, p = 0.533, genotype×gender: F_1, 59_ = 0.99, p = 0.324). Similar levels of freezing behavior for auditory cues were found between GPR55 KO and littermate control mice (genotype: F_1, 59_ = 1.723, p = 0.194, gender: F_1, 59_ = 1.34, p = 0.252, genotype×gender: F_1, 59_ = 0.383, p = 0.538). Thus, GPR55 doesn’t seem to be critical for the learning and memory of cue- or context-dependent fear-conditioning.

## Discussion

GPR55 has been implicated in diverse biological processes including analgesia [11], oncogenesis [13], and bone growth [12]. Given the widespread expression of GPR55 in the brain, and due to the lack of specific GPR55 agonists and antagonists, we have explored the CNS roles of GPR55 using mice lacking this receptor. GPR55 KO mice develop normally without any gross defects in brain structures. The absence of GPR55 did not significantly affect the abundance of endocannabinoids and related lipids or mRNA levels for components of the endocannabinoid system in the hippocampus. Using an array of behavioral assays, we found that GPR55 KO mice have deficits in motor coordination and thermal sensitivity. In contrast, GPR55 and their WT littermates appear indistinguishable from one another in measures of anxiety/depression, sensory-motor gating and fear conditioning behaviors. In addition, hippocampal synaptic transmission, short-term synaptic plasticity, and long-term potentiation were similar in WT and GPR55 KO mice. These results suggest that GPR55 is likely involved in a fairly narrow range of behaviors and that GPR55-directed therapies may have few adverse CNS side effects.

Motor coordination is controlled by various brain regions, including the motor cortex, basal ganglia, and cerebellum [45], [46], [47]. Our qRT-PCR studies found that GPR55 is most abundant in the striatum and is less prevalent in the cerebellum. Using the rotarod assay, we found that GPR55 KO mice consistently performed worse than WT littermates with reduced latency to fall from rotating drum across repeated trials over two days. Nonetheless, GPR55 KO mice were able to significantly increase the amount of time on the accelerating rotarod upon repeated trials. Thus, despite the initial deficit, GPR55 KO mice appear to learn this motor task in a similar fashion as WT mice. The significant increase in the number of footslip errors in the GPR55 KO mice, but not in walking distance during parallel rod assays provide additional support for a role of GPR55 in motor coordination.

The regulation of Purkinje cell activity is important for motor behavior and motor learning [51]. Multiple forms of endocannabinoid-mediated retrograde modulation are found in both excitatory and inhibitory synapses in the cerebellar cortex ([52], [53], [54], [55], [56], [57], reviewed in [51]). The most abundant cannabinoid receptor in cerebellum is the CB1R [58], and it has been linked directly to cerebellum-dependent motor learning in the delayed eyeblink conditioning assay [59]. Whether GPR55 plays a role in modulating cerebellar Purkinje cells function and whether cerebellar GPR55 relates to motor coordination deficits remain to be determined.

Muscle strength, motivation to move, vestibular dysfunction, proprioceptive feedback, as well as individual differences in anxiety can potentially contribute to the deficits in the rotarod and parallel rod assays [48], [60]. The normal behaviors for GPR55 KO mice in the inverted screen, wire-hang, grip strength and dowel assays suggest that GPR55 KO mice have normal gross motor skills and muscle strength. While the motor function deficit seen on the rotarod test could be caused by novelty-induced anxiety when mice are introduced to the apparatus, GPR55 KO mice showed no signs of anxiety in either the elevated plus maze or open field assays, making this explanation unlikely.

Mice with a perturbation in the auditory function often display vestibular impairment in addition to hearing loss [61]. To explore the possibility of vestibular impairment in GPR55 KO mice, we subjected GPR55 KO and WT littermates to the swim test [61]. Mutant mice with vestibular abnormality display irregular swimming pattern including vertical swimming, swimming in a circle, swimming on side (left or right preference), or swimming in an unbalanced manner (tail is raised and beats around in an unstable manner) [61], [62], [63]. We did not observe an increase in abnormal swimming patterns in the GPR55 KO mice (data not shown). This observation suggests that GPR55 KO mice have normal vestibular function. In addition, PPI was equally suppressed by lower intensity tones suggesting a normal sensory response to that level of auditory stimulation in GPR55 KO mice.

The regional distribution of GPR55 in the brain is controversial. Using quantitative PCR we found that GPR55 mRNA was expressed in the following order (from high to low abundance): striatum>hippocampus>forebrain>cortex>cerebellum, whereas in a previous report the expression levels were found in the following order: frontal cortex>striatum>hippocampus = cerebellum [5]. The major difference between our studies is the abundance of GPR55 in the cortical area and hippocampus. In agreement with our findings, human GPR55 mRNA was found at the highest levels in the basal ganglia (striatum, caudate nucleus and putamen), followed by nucleus accumbens, hypothalamus, hippocampus, and other brain regions, with lowest expression levels in the cerebellum [64]. It should be emphasized that the abundance of a receptor does not necessarily correlate to its importance in a given behavior.

Staton et al. [11] showed that loss of GPR55 perturbs thermal nociception and found that GPR55 knockout mice failed to develop mechanical hyperalgesia following treatment with Freund’s adjuvant or partial nerve ligation. In the present study we also found mild perturbation of thermal nociception (Table 2) in GPR55 knockout mice. The presence of high levels of GPR55 in large diameter dorsal root ganglion (DRG) neurons compared to small diameter DRG neurons is consistent with subtle effects on acute nociceptive responses. Based on previous finding that chronic inflammation or long-standing nerve injury will recruit large diameter DRG neurons into nociceptive pathways [65], [66], it will be important to investigate differences between wildtype and GPR55 knockout mice in chronic pain models.

The GPR55 knockout mice used in Staton et al. [11] were made by deleting amino acids between residues 38 and 282 (corresponding to the first through part of the seventh transmembrane domain). Hence the first 118 bp of GPR55 as well as the distal portion of the sequence beyond lysine 281 are left intact in this strain. This could lead to an incomplete removal of the GPR55 gene fragment as this latter sequence contains several methionines, making it possible to be translated if it is transcribed. The TIGM GPR55 mice used in this study were generated by deleting exon 2 of the *Gpr55* gene. This exon contains the entire coding region of GPR55 protein. The absence of GPR55 transcript in GPR55 KO brains of the line used in this study confirmed the complete removal of GPR55 function [22].

Two very recent brief reports found that GPR55 modulates synaptic strength at Schaffer collateral/CA1 synapses during period of high synaptic activity [67], [68]. Thus, it is interesting to note that a task involving the hippocampus (contextual fear conditioning), as well as several measures of hippocampal synaptic activity (DSE, membrane excitability, synaptic strength, and LTP) were indistinguishable between GPR55 KO and WT mice. This may mean that GPR55 fills a specialized role in modulating hippocampal circuits that is not evident in the behavioral and physiological assays used in the current study.

Most preclinical studies of GPR55 suggest that GPR55 antagonists might be therapeutically beneficial. For example, GPR55 deletion ablates hyperalgesia following partial nerve ligation and injection of complete Freund’s adjuvant [11] and activation of GPR55 promotes cancer cell proliferation and GPR55 expression levels positively correlate with tumor aggressiveness across multiple tumor types [13], [20]. Extrapolating from knockout studies to receptor antagonist action must be done carefully, but the results of the current study suggest that antagonism of GPR55 will be associated with mild CNS side effects and that investigation of possible adverse effects should particularly focus on those involving motor coordination.
